# Obstructive sleep apnea screening in different age groups: performance of the Berlin, STOP-Bang questionnaires and Epworth Sleepiness Scale

**DOI:** 10.1016/j.bjorl.2023.101283

**Published:** 2023-06-28

**Authors:** Daniel Villela e Silva, Davi da Silveira Barroso Alves, Mariana da Rocha Rodrigues Nóbrega, Felipe Braga Coelho Gomes Ribeiro, Leandro Yukio Yatabe Franco, Isadora Rodrigues da Silva, Lucia Joffily, Maira da Rocha, Paulo Henrique Godoy

**Affiliations:** a Universidade Federal do Estado do Rio de Janeiro, Rio de Janeiro, RJ, Brazil; b Laboratório do Sono da Universidade Federal do Estado de Rio de Janeiro (LABSONO-UNIRIO), Rio de Janeiro, RJ, Brazil; c Universidade Federal do Estado do Rio de Janeiro, Departamento de Métodos Quantitativo, Rio de Janeiro, RJ, Brazil; d Hospital Universitário Gaffrée e Guinle (HUGG), Empresa Brasileira de Serviços Hospitalares (EBSERH), Rio de Janeiro, RJ, Brazil

**Keywords:** Sleep apnea, Age groups, Surveys and questionnaires, Diagnostic techniques and procedures, Polysomnography

## Abstract

**Objective:**

To evaluate the performance of the Berlin Questionnaire, the STOP-Bang Questionnaire, and the Epworth Sleepiness Scale in screening for the disease in adults of different age groups by comparing them with polysomnography.

**Methods:**

Cross-sectional study with prospective patient allocation, in which individuals underwent a medical interview, completion of the three screening instruments, and polysomnography. Individuals were categorized into three age groups: 18–39, 40–59, and ≥60 years. The results of the screening instruments were compared to the diagnostic criteria of the International Classification of Sleep Disorders—third edition. Performance was assessed using 2 × 2 contingency tables, estimating sensitivity, specificity, predictive value, likelihood ratio, and accuracy. Receiver Operating Characteristic curves were also constructed and the area under the curve was estimated for each instrument by age group.

**Results:**

We obtained a sample with 321 individuals suitable for analysis. The mean age was 50 years, with a predominance of females (56%). The prevalence of the disease in the overall sample was 79%, more prevalent in males in any age group and more frequent in the middle age group. The analyzes revealed that STOP-Bang performed better, both for the overall sample and for all age groups, followed by Berlin Questionnaire and Epworth Sleepiness Scale.

**Conclusion:**

In an outpatient setting with individuals with characteristics similar to those in this study, it seems sensible to choose the STOP-Bang as a screening tool for the disease, regardless of age group.

**Level of evidence according the guide for authors:**

level 2.

## Highlights

• Performance of screening instruments for obstructive sleep apnea.
• Obstructive sleep apnea screening in different age groups.
• The STOP-Bang questionnaire has high sensitivity for screening OSA.
• ROC curve and area under the curve of three sleep disorder screening instruments.
• Age has no significant influence on the screening for obstructive sleep apnea.

## Introduction

Obstructive Sleep Apnea (OSA) is a very prevalent respiratory disorder, affecting 32.8% of the general population.^1^ Its pathophysiology results from the interaction of several factors, such as sleep physiology, nasal airflow,^2^ respiratory control and muscle control of the upper airway,^3^ which are influenced by age,^4^ making it difficult to identify just one site or cause of obstruction.^5^ A trend of increased prevalence is observed in the general population, possibly due to factors such as population aging, reaching approximately 84% between the ages of 60 and 85.^6^ Young and old people may have a different presentation of the disease,^7^ and the latter tend to present mild or unusual symptoms of the disease, such as mood swings, insomnia, and cognitive impairment,^1^ making it even more difficult to suspect the presence of the disease and, consequently, recommend type 1 Polysomnography (PSG). This test, which is the gold standard test for OSA diagnosis, has high costs and low availability, especially in middle and low-income countries.^9^ Thus, given the difficult access to PSG, clinical instruments for OSA screening have gained increasing importance in clinical practice, helping in a standardized assessment and in the more accurate selection of individuals who should undergo PSG.^10^ These instruments present different performances depending on the characteristics of the population studied, whether elderly, sleep laboratory patients, surgical patients or those who suffer from a comorbidity.^11^, ^12^ Among the instruments already validated and widely used, we have the Berlin (BQ)^13^, ^14^ and STOP-Bang (SBQ) Questionnaires,^11^, ^15^ as well as the Epworth Sleepiness Scale (ESS),^16^, ^17^ which, despite not having been developed specifically for OSA investigation,^16^ ends up aiding in screening for OSA, since it assesses excessive daytime sleepiness, one of the symptoms presented by some OSA patients. In this context, the aim of this study was to evaluate the performance of the BQ, SBQ, and the ESS in screening for obstructive sleep apnea syndrome in adults of different age groups, comparing them to the results of the gold standard, type 1 polysomnography.

## Methods

This is a cross-sectional study, with prospective allocation of patients, conducted between February 2020 and January 2022. The sample consisted of individuals from various clinical specialties and seen at the Outpatient Sleep Medicine Clinic of a University Hospital. Inclusion criteria were individuals aged ≥18 years. Those who were unable to adequately understand and answer the questions posed by the examiner without the help of a third party, had a previous history of OSA-related tests, had PSG of poor technical quality with an examination time <4 h, or with a prevalence of central events, had incomplete interview data collected, or had a previous diagnosis of OSA were excluded. The study protocol complied with the Helsinki Declaration and was approved by the Research Ethics Committee, through Plataforma Brazil, substantiated opinion number 3.298.539. Informed consent was obtained from all participants.

An interview was conducted with collection of sociodemographic and clinical-epidemiological data and application of the BQ, SBQ and ESS. In a second stage, all underwent PSG, with registration time and technical quality considered adequate for analysis.

The sample was categorized into 3 age groups: 18–39 years, 40–59 years, and 60 years or older. Each group was analyzed regarding the presence or absence of OSA.

### Screening instruments

For the application of the questionnaires, the criteria of the versions approved for use in Brazil were followed. For the BQ, patients with two or more positive categories were classified as high risk for OSA.^18^ For the SBQ, those who presented scores ≥3 were classified as high risk for the disease.^19^ For the ESS, individuals with scores >10 were considered to have probable excessive daytime sleepiness.^17^

### Gold-standard

All PSG were performed at the Sleep Laboratory of the Federal University of the State of Rio de Janeiro (LABSONO-UNIRIO). The EMSA system, approved by the Brazilian National Health Surveillance Agency (ANVISA), was used, and had a complete record (more than 7 channels). The electroencephalogram, electrooculogram, chin and leg electromyogram, nasal airflow, thermistor, thoracic and abdominal plethysmography, snoring sensor, body position sensor, pulse oximeter with oxyhemoglobin saturation recording, and electrocardiogram were monitored continuously and simultaneously. In order to maintain a standard of interpretation, the records were manually recorded according to the American Academy of Sleep Medicine (AASM)^20^ criteria by trained physicians, all belonging to the LABSONO clinical staff. They were blind as to the results obtained in the screening instruments. The investigator who performed the statistical analyzes was also blinded. The tests with a duration of more than 4 h and without relevant interpretation interference were considered suitable for analysis. Exams with a predominance of central events were not considered.

### Diagnosis

The diagnostic criteria followed the International Classification of Sleep Disorders-third edition (ICSD-3),^21^ without considering the degree of intensity/severity of the disease. To be classified as having OSA, it was necessary to present signs, symptoms, or comorbidities correlated to OSA and an Apnea-Hypopnea Index (AHI) ≥5 events/h, or only AHI ≥ 15 events/h.

### Statistical analysis

The screening instruments were analyzed for OSA identification in relation to PSG results, for each age group.

For the performance analysis of QB, QSB, and ESE, 2 × 2 contingency tables were used, and sensitivity, specificity, Positive and negative Predictive Value (PPV) and Negative Predictive Value (NPV), Positive likelihood Ratio (PSR) and Negative Likelihood Ratio (NLR), and accuracy, with 95% Confidence Interval, were estimated in each age group.

Receiver Operating Characteristic (ROC) curves were constructed and Areas Under the Curve (AUC) were calculated for each screening instrument by age group. Descriptive measures of the score of each questionnaire according to the diagnosis of OSA were obtained for each age group. The Shapiro-Wilks test was applied to verify the distribution of the scores. For distributions that were not normal, the Wilcoxon test was used to assess differences in the medians between patients with and without the diagnosis of OSA. The AUCs obtained were compared two by two using DeLong's test.^22^ The curves and AUC comparison tests were performed using the pROC package of the R program.^23^

## Results

From a total of 494 individuals seen and interviewed, 173 were excluded according to the criteria. Thus, 321 patients were considered suitable for analysis, and the following distribution by age was observed: 83 patients (25.9%) aged 18–39 years, 148 (46.1%) aged 40–59 years, and 90 (28.0%) aged 60 years or more.

We found 254 (79.1%) individuals with OSA in the overall sample, and the mean age was slightly higher among those with OSA (51 years) compared to the mean age of those without the disease (48 years).

Even with the predominance of females in the overall sample (56%), we observed a higher number of males with OSA compared to those without the disease when compared to females. The gender variable showed significant difference (Table 1).

**Table 1:** Sociodemographic and clinical-epidemiological data according to the presence of obstructive apnea in the general sample and by age groups.

Variables	General sample (*n* = 321)		18–39 years old (*n* = 83)		40–59 years old (*n* = 148)		60 years and older (*n* = 90)	
	OSA		OSA		OSA		OSA	
	Yes	No	Yes	No	Yes	No	Yes	No
	254	67	56	27	127	21	71	19
Sex	*p*-value < 0.001		*p*-value = 0.005		*p*-value = 0.03		*p*-value = 0.090	
Male	123 (48%)	17 (25%)	37 (66%)	9 (33%)	56 (44%)	4 (19%)	30 (42%)	4 (21%)
Female	131 (52%)	50 (75%)	19 (34%)	18 (67%)	71 (56%)	17 (81%)	41 (58%)	15 (79%)
Age	*p*-value = 0.054		*p*-value = 0.003		*p*-value > 0.9		*p*-value = 0.4	
	51	48	30	26	49	49	65	65
Civil Status	*p*-value = 0.006		*p*-value = 0.8		*p*-value = 0.2		*p*-value = 0.15	
Single	72 (28%)	35 (52%)	36 (64%)	19 (70%)	27 (21%)	9 (43%)	9 (13%)	7 (37%)
Married/Living together	140 (55%)	26 (39%)	18 (32%)	8 (30%)	84 (66%)	11 (52%)	38 (54%)	7 (37%)
Separated/Divorced	28 (11%)	5 (7.5%)	2 (3.6%)	0 (0%)	13 (10%)	1 (4.8%)	13 (19%)	4 (21%)
Widowed	13 (5.1%)	1 (1.5%)	0 (0%)	0 (0%)	3 (2.4%)	0 (0%)	10 (14%)	1 (5.3%)
Education	*p*-value = 0.4		*p*-value = 0.4		*p*-value > 0.9		*p*-value > 0.9	
	12 (9, 15)	12 (11, 15)	13 (11, 16)	13 (12, 15.5)	11 (9, 15)	12 (9, 14)	11 (7, 15)	11 (6.5, 14)
Drinking	*p*-value = 0.6		*p*-value > 0.9		*p*-value = 0.6		*p*-value = 0.5	
Yes	21 (8.3%)	3 (4.5%)	8 (14%)	3 (11%)	7 (5.5%)	0 (0%)	6 (8.6%)	0 (0%)
No	232 (92%)	64 (96%)	48 (86%)	24 (89%)	120 (94%)	21 (100%)	64 (91%)	19 (100%)
Tabacco Use	*p*-value = 0.4		*p*-value = 0.7		*p*-value = 0.3		*p*-value = 0.088	
Yes	24 (9.5%)	10 (15%)	5 (8.9%)	1 (3.7%)	13 (10%)	4 (19%)	6 (8.6%)	5 (26%)
No	229 (91%)	57 (85%)	51 (91%)	26 (96%)	114 (90%)	17 (81%)	64 (91%)	14 (74%)
Exercise	*p*-value > 0.9		*p*-value > 0.9		*p*-value = 0.6		*p*-value = 0.8	
Yes	58 (23%)	16 (24%)	16 (29%)	7 (26%)	27 (21%)	6 (29%)	15 (21%)	3 (16%)
No	194 (77%)	51 (76%)	39 (71%)	20 (74%)	100 (79%)	15 (71%)	55 (79%)	16 (84%)
Type 2 DM	*p*-value = 0.2		*p*-value = 0.5		*p*-value = 0.4		*p-*value = 0.2	
Yes	50 (20%)	9 (13%)	3 (5.4%)	0 (0%)	20 (16%)	5 (24%)	27 (38%)	4 (21%)
No	204 (80%)	58 (87%)	53 (95%)	27 (100%)	107 (84%)	16 (76%)	44 (62%)	15 (79%)
Hypertension	*p*-value = 0.084		*p*-value = 0.014		*p*-value = 0.8		*p*-value = 0.9	
Yes	121 (48%)	24 (36%)	11 (20%)	0 (0%)	63 (50%)	11 (52%)	47 (66%)	13 (68%)
No	133 (52%)	43 (64%)	45 (80%)	27 (100%)	64 (50%)	10 (48%)	24 (34%)	6 (32%)
CAD	*p*-value = 0.3		*p*-value = 0.5		*p*-value = 0.4		*p*-value > 0.9	
Yes	22 (8.7%)	3 (4.5%)	1 (1.8%)	1 (3.7%)	11 (8.7%)	0 (0%)	10 (14%)	2 (11%)
No	232 (91%)	64 (96%)	55 (98%)	26 (96.3%)	116 (91%)	21 (100%)	61 (86%)	17 (89%)
Heart Failure	*p*-value = 0.5		*p*-value = 0.3		*p*-value > 0.9		*p*-value > 0.9	
Yes	6 (2.4%)	1 (1.5%)	0 (0%)	1 (3.7%)	0 (0%)	0 (0%)	6 (8.5%)	0 (0%)
No	248 (97.6%)	66 (98.5%)	56 (100%)	26 (96.3%)	127 (100%)	21 (100%)	65 (91.5)	19 (100%)
Atrial Fibrillation	*p*-value > 0.9		*p*-value > 0.9		*p*-value > 0.9		*p*-value > 0.9	
Yes	2 (0.8%)	0 (0%)	1 (1.8%)	0 (0%)	1 (0.8%)	0 (0%)	0 (0%)	0 (0%)
No	252 (99.2%)	67 (100%)	55 (98%)	27 (100%)	126 (99%)	21 (100%)	71 (100%)	19 (100%)
CVA	*p*-value > 0.9		*p*-value > 0.9		*p*-value > 0.9		*p*-value > 0.9	
Yes	1 (0.4%)	0 (0%)	0 (0%)	0 (0%)	1 (0.8%)	0 (0%)	0 (0%)	0 (0%)
No	253 (100%)	67 (100%)	56 (100%)	27 (100%)	126 (99%)	21 (100%)	71 (100%)	19 (100%)
COPD	*p*-value = 0.2		*p*-value > 0.9		*p*-value = 0.14		*p*-value > 0.9	
Yes	0 (0%)	1 (1.5%)	0 (0%)	0 (0%)	0 (0%)	1 (4.8%)	0 (0%)	0 (0%)
No	254 (100%)	66 (99%)	56 (100%)	27 (100%)	127 (100%)	20 (95%)	71 (100%)	19 (100%)
Asthma	*p*-value = 0.4		*p*-value > 0.9		*p*-value = 0.3		*p*-value = 0.3	
Yes	5 (2.0%)	3 (4.5%)	1 (1.8%)	0 (0%)	1 (0.8%)	1 (4.8%)	3 (4.2%)	2 (11%)
No	249 (98%)	64 (96%)	55 (98%)	27 (100%)	126 (99%)	20 (95%)	68 (96%)	17 (89%)
Reflux	*p*-value = 0.14		*p*-value = 0.3		*p*-value = 0.5		*p*-value = 0.062	
Yes	41 (16%)	6 (9.0%)	7 (12%)	1 (3.7%)	21 (17%)	5 (24%)	13 (18%)	0 (0%)
No	213 (84%)	61 (91%)	49 (88%)	26 (96.3%)	106 (83%)	16 (76%)	58 (82%)	19 (100%)
CRF	*p*-value = 0.2		*p*-value > 0.9		*p*-value > 0.9		*p*-value = 0.2	
Yes	0 (0%)	1 (1.5%)	0 (0%)	0 (0%)	0 (0%)	0 (0%)	0 (0%)	1 (5.3%)
No	254 (100%)	66 (99%)	56 (100%)	27 (100%)	127 (100%)	21 (100%)	71 (100%)	18 (95%)

*p*-value, Significance level; OSA, Obstructive Sleep Apnea; DM, Diabetes Mellitus; CAD, Chronic Arterial Disease; CVA, Cerebrovascular Accident; COPD, Chronic Obstructive Pulmonary Disease; CRF, Chronic Renal Failure.

**Figure 1:**
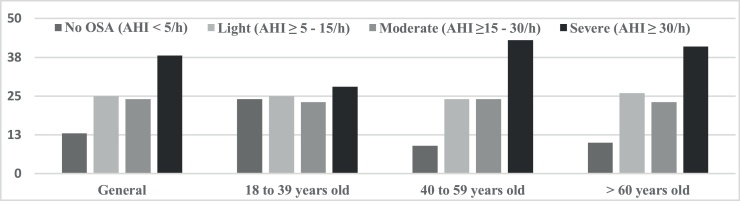
Presence and degree of obstructive sleep apnea for the overall sample and age groups. OSA, Obstructive Sleep Apnea; AHI, Apnea-Hypopnea Index.

Using only the PSG results as criteria (AHI ≥5 events/h), OSA was identified in 87% of the patients in the overall sample. Regarding degree of severity of the disease, the highest percentage was concentrated in the severe degree. The mild degree had the second highest frequency of individuals, with little percentage difference in the overall sample or in the age groups (Fig. 1).

**Figure 2:**
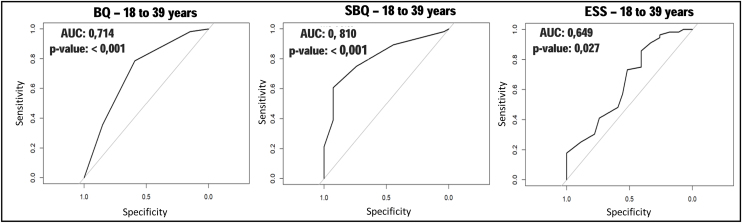
ROC curves for the Berlin Questionnaire, STOP-Bang and Epworth Sleepiness Scale in subjects aged 18–39 years. *p*-value, significance level; AUC, Area Under the Curve; BQ, Berlin Questionnaire; SBQ, STOP-Bang Questionnaire; ESS, Epworth Sleepiness Scale.

As for performance of the instruments, in the general sample, the SBQ stood out for presenting higher sensitivity, PPV, SVR and accuracy (Table 2). In the 18–39 age group, the highest sensitivity of the BQ stood out, but the SBQ showed higher PPV, SVR and accuracy values. The ESS, however, had the worst performance. When analyzing the ROC curves with their respective AUC, it was observed that the SBQ presented the highest AUC (Fig. 2). However, when compared to BQ, it showed little advantage, though statistically significant (*p* =  0.04426). The ESS obtained the worst AUC and, when compared to the BQ, there was no significant difference between them (*p* =  0.3157).

**Table 2:** Performance of the Berlin, STOP-Bang and Epworth Sleepiness Scale questionnaires compared to type 1 polysomnography in the general sample and by age groups.

Instrument	General Sample (*n* = 321)	18–39 years old (*n* = 83)	40–59 years old (*n* = 148)	60 years old or older (*n* = 90)
BQ				
Instrument/PSG	75%/79%	67%/67%	82%/86%	70%/79%
Sens	0.81 (0.76, 0.86)	0.80 (0.67, 0.90)	0.85 (0.78, 0.91)	0.75 (0.63, 0.84)
Spec	0.48 (0.35, 0.60)	0.59 (0.39, 0.78)	0.33 (0.15, 0.57)	0.47 (0.24, 0.71)
PPV	0.85 (0.80, 0.90)	0.80 (0.67, 0.90)	0.89 (0.81, 0.94)	0.84 (0.73, 0.92)
NPV	0.40 (0.29, 0.52)	0.59 (0.39, 0.78)	0.27 (0.12, 0.48)	0.33 (0.17, 0.54)
PLR	1.55 (1.22, 1.97)	1.96 (1.22, 3.15)	1.28 (0.93, 1.74)	1.42 (0.91, 2.22)
NSR	0.40 (0.28, 0.57)	0.34 (0.18, 0.62)	0.45 (0.22, 0.93)	0.54 (0.29, 0.99)
Accur	0.74 (0.69, 0.79)	0.73 (0.62, 0.82)	0.78 (0.70, 0.84)	0.69 (0.58, 0.78)
SBQ				
Instrument/PSG	78%/79%	59%/67%	86%/86%	70%/79%
Sens	0.86 (0.81, 0.90)	0.75 (0.62, 0.86)	0.89 (0.82, 0.94)	0.89 (0.79, 0.95)
Spec	0.52 (0.40, 0.65)	0.74 (0.54, 0.89)	0.33 (0.15, 0.57)	0.42 (0.20, 0.67)
PPV	0.87 (0.82, 0.91)	0.86 (0.73, 0.94)	0.89 (0.82, 0.94)	0.85 (0.75, 0.92)
NPV	0.49 (0.37, 0.61)	0.59 (0.41, 0.75)	0.33 (0.15, 0.57)	0.50 (0.25, 0.75)
PLR	1.80 (1.39, 2.32)	2.89 (1.50, 5.57)	1.33 (0.98, 1.82)	1.53 (1.04, 2.27)
NSR	0.27 (0.19, 0.40)	0.34 (0.20, 0.56)	0.33 (0.15, 0.72)	0.27 (0.12, 0.62)
Accur	0.79 (0.74, 0.83)	0.75 (0.64, 0.84)	0.81 (0.74, 0.87)	0.79 (0.69, 0.87)
ESS				
Instrument/PSG	50%/79%	65%/67%	52%/86%	31%/79%
Sens	0.54 (0.47, 0.60)	0.73 (0.60, 0.84)	0.54 (0.45, 0.63)	0.37 (0.25, 0.49)
Spec	0.66 (0.53, 0.77)	0.52 (0.32, 0.71)	0.62 (0.38, 0.82)	0.89 (0.67, 0.99)
PPV	0.86 (0.79, 0.91)	0.76 (0.62, 0.87)	0.90 (0.81, 0.95)	0.93 (0.76, 0.99)
NPV	0.27 (0.20, 0.35)	0.48 (0.29, 0.67)	0.18 (0.10, 0.29)	0.27 (0.17, 0.40)
PLR	1.56 (1.10, 2.21)	1.52 (1.00, 2.32)	1.43 (0.81, 2.52)	3.48 (0.91,13.37)
NSR	0.71 (0.57, 0.88)	0.52 (0.29, 0.91)	0.74 (0.50, 1.08)	0.71 (0.56, 0.90)
Accur	0.56 (0.50, 0.62)	0.66 (0.55, 0.76)	0.55 (0.47, 0.64)	0.48 (0.37, 0.59)

PSG, Polysomnography type 1; BQ, Berlin Questionnaire; SBQ, STOP-Bang Questionnaire; ESS, Epworth Sleepiness Scale; Sens, Sensitivity; Spec, Specificity; PPV, Positive Predictive Value; NPV, Negative Predictive Value; PLR, Positive Likelihood Ratio; NSR, Negative Likelihood Ratio; Accur, Accuracy.

**Figure 3:**
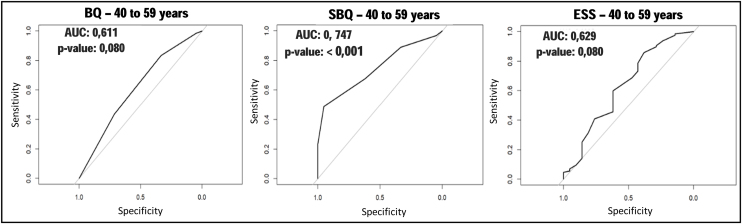
ROC curves, for the Berlin Questionnaire, STOP-Bang and Epworth Sleepiness Scale in subjects aged 40–59 years. *p*-value, significance level; AUC, Area Under the Curve; BQ, Berlin Questionnaire; SBQ, STOP-Bang Questionnaire; ESS, Epworth Sleepiness Scale.

Among those aged 40–59 years, the SBQ maintained the best values for sensitivity and accuracy, whereas ESS presented the highest specificity, PPV and SVR (Table 2). As for AUC, the SBQ maintained the best performance, among the three instruments, while ESS presented the worst value (Fig. 3). As in the previous age group, it was observed that the BQ and SBQ presented a significant difference in the AUC (*p* = 0.01123), with the latter performing better. This difference was not observed between the BQ and ESS (*p* =  0.8177), as between the SBQ and ESS (*p* =  0.06509).

**Figure 4:**
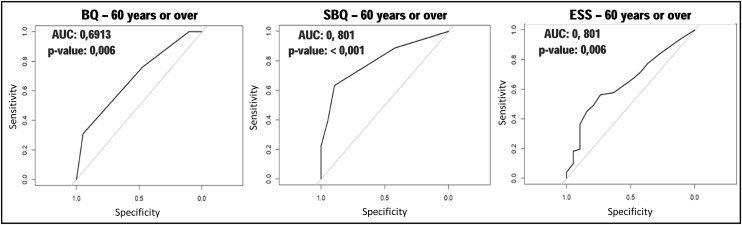
ROC curves, for the Berlin Questionnaire, STOP-Bang and Epworth Sleepiness Scale in individuals aged 60 years and older. *p*-value, significance level; AUC, Area Under the Curve; BQ, Berlin Questionnaire; SBQ, STOP-Bang Questionnaire; ESS, Epworth Sleepiness Scale.

Among individuals ≥60 years, the SBQ also had good sensitivity, but the ESS stood out for the highest specificity values, PPV and SVR, with the latter more than double the result from the other two instruments (Table 2). Regarding ROC curves and AUC, the results remained similar to the other age groups. The SBQ presented the best performance, with the highest AUC (Fig. 4), followed by the BQ and ESS, however, the difference in performance between these last two instruments was not significant (*p* =  0.6187).

## Discussion

The motivation of this study was to analyze the three most commonly used clinical instruments in OSA screening in order to verify whether the individual's age range would influence their performance. This is justified by the fact that individuals with OSA may present different signs, symptoms and clinical implications of the disease depending on their age,^7^, ^8^ which is not predicted or adjusted in the BQ, SBQ or ESS.

Given the difficulty of access to PSG,^9^ the use of these instruments takes on even greater importance, functioning as screening to be applied before a more costly and complex exam is requested.

Thus, we also tried to identify if there would be, among the three instruments, any that would perform better in OSA screening, depending on the age group. It is expected that an ideal screening instrument would have high sensitivity and reasonable specificity.^24^ As observed in results, the SBQ had a good performance in all age groups, with good sensitivity among those ≥40 years. However, in a clinical investigation, predictive values gain importance for estimating the probability of disease (or its absence) from the test result,^24^ while the likelihood ratio contributes in estimating how much a given test contributes to the probability of disease detection, compared to the prevalence of this disease.^25^ The SBQ also showed good values for PSR and PPV, but the latter was also high for the other two instruments, probably due to the high prevalence of OSA in the sample.

The construction of the ROC curves and calculation of the AUC provided a simpler and more objective analysis between the instruments, corroborated the tests cited and added information on the comparability between the instruments in the different age groups. It was evident, for example, that between 18 and 39 years of age, the SBQ was the instrument with the best results, but with little difference compared to the BQ. Thus, in this age group, both instruments could be used satisfactorily as screening tests, unlike the ESS, which showed a non-significant AUC value in this age group.

Among individuals aged 40–59 years, the SBQ also presented the highest AUC value, being the only instrument with a *p* value lower than 0.05. However, a significant difference was observed between the AUC values of BQ and SBQ. This may be justified by the fact that the ESS presented the best PPV and specificity values among the instruments in this age group.

Among individuals aged 60 years or older, once again the SBQ showed better performance. The comparison between BQ and ESS did not show a significant *p*-value, and as such, in this age group, we could not conclude which, between these two, had a better performance. It is worth noting that ESS presented its highest AUC value, with *p* <  0.05. This may be explained by its high specificity and PLR in this age group.

This better overall performance of the SBQ had already been evidenced by other researchers, in samples with similar mean ages as the present study,^25^, ^26^ in which SBQ showed the highest and most significant AUC in all age groups. On the other hand, the BQ only showed significant AUC for individuals between the ages of 18 and 39. The good performance of these two questionnaires among younger people may be explained by the fact that they present sub-items that assess the presence of comorbidities and anthropometric data, which have a strong association with OSA in this age group.^25^, ^27^, ^28^

The ESS, however, assesses symptoms connected to daytime sleepiness without considering comorbidities or anthropometric data. Among individuals aged 60 years and older, although the SBQ still maintained the best performance, the ESS showed its highest AUC value, significant only in this age group. This may be explained by the fact that many elderly people without sleep disorders nap due to the absence of socially imposed schedules, such as those related to work or education, which is not necessarily indicative of pathological daytime sleepiness.^29^

Thus, even though this study does not have external validity, because it is unicentric and carried out with individuals seen in a sleep laboratory, there is the perception that the SBQ is the instrument with the best overall performance. Its application can bring benefits to the population, since it is a practical, short instrument with a simple and direct scoring system, and can be applied by any medical specialty or in family medicine teams.^19^

The present study had some limitations: the patients analyzed were those referred to or who voluntarily sought care in the HUGG sleep laboratory, which increases the chance of selection bias. Being a single-center study limits the implications of the results for the general population. On the other hand, among the strengths of this study, one can highlight the considerable sample of adults, all of whom underwent complete polysomnography, performed in a center that is a reference for the diagnosis and treatment of sleep-related disorders, with manual analysis of the results by physicians’ blind to the scores of the instruments. There are few studies in the literature that evaluate and compare the performance of OSA screening instruments in different age groups.

## Conclusions

In conclusion, the present study, carried out with adult individuals investigated in a Sleep Laboratory with a prevalence of OSA similar to that observed in the literature, showed that the Berlin and STOP-Bang questionnaires performed well in recognizing obstructive sleep apnea syndrome. The most noteworthy was the STOP-Bang, which performed well for any age group. The Epworth Sleepiness Scale did not prove to be a good option for tracking the disease in the sample studied, regardless of age range.

Although the present study has no external validation, it seems sensible to consider applying the STOP-Bang questionnaire, within a clinical setting, without the concern that age may significantly influence the screening method for obstructive sleep apnea. This consideration would be particularly valid in individuals with a similar profile to those investigated in the present study.

## Funding

The project that originated this study received funding for research from the Federal University of the State of Rio de Janeiro (UNIRIO) through Dean of Graduate Studies, Research and Innovation (PROPGPI) and the Research Board (DPq), Edital Programa Pesquisador Instalação UNIRIO – PROPGPI (PPINST/DPq-UNIRIO) 2019.

The present study was the subject of a project for the master’s degree of studies at the Postgraduate Degree in Neurology of the Federal University of the State of Rio de Janeiro.

## Conflicts of interest

The authors declare no conflicts of interest.
